# Analysis of Fish Diversity and Invasive Species in the Cuijiang River Based on Environmental DNA Metabarcoding

**DOI:** 10.3390/biology15151276

**Published:** 2026-08-03

**Authors:** Haiqi Li, Liming Shao, Daizhong Huang, Yi Zhou, Haipeng Guo, Lujing Sang, Zhi Zhang, Lingjun Xiao, Wuhui Li, Liang Guo, Shengnan Li, Chongqing Wang, Kaikun Luo, Zhongyuan Shen

**Affiliations:** 1 Engineering Research Center of Polyploid Fish Reproduction and Breeding of the State Education Ministry, College of Life Sciences, Hunan Normal University, Changsha 410081, Chinasanglujing2021@163.com (L.S.);; 2 Hunan Provincial Dongting Lake Ecological Environment Monitoring Center, Yueyang 414000, China; 3 Fujian Key Laboratory of Conservation and Sustainable Utilization of Marine Biodiversity, College of Geography and Oceanography, Minjiang University, Fuzhou 350108, China; zhizhang@mju.edu.cn; 4 Wuhan Angelwing CDR Technology Co., Ltd., Wuhan 430000, China

**Keywords:** eDNA Metabarcoding, fish diversity, invasive alien species, Cuijiang River

## Abstract

eDNA metabarcoding provides distinct benefits—including non-invasiveness, cost-effectiveness, and high detection sensitivity—that make it particularly appropriate for aquatic ecosystem conservation and fisheries management. An environmental DNA (eDNA) metabarcoding survey was performed in the Cuijiang River to assess the feasibility of this technique for fish diversity monitoring. Fish diversity was evaluated through water collection, eDNA extraction and purification, and subsequent bioinformatics analysis. Our results revealed that the surveyed river section harbours at least 36 fish species, belonging to 30 genera, 13 families, and 6 orders. The fish community was numerically dominated by Cypriniformes. Notably, invasive alien species represented a relatively high proportion (24.72%) of the total operational taxonomic units (OTUs). Alpha diversity indices showed spatial heterogeneity among the monitoring sites, which is likely influenced by anthropogenic activities, land-use patterns, and hydrological connectivity in the sampling areas. Overall, eDNA metabarcoding proves to be an effective approach for fish diversity assessment and can serve as a valuable supplementary tool to conventional methods, with particular utility for the early detection of elusive and invasive species. We therefore recommend its incorporation into ongoing and future aquatic monitoring and conservation frameworks to strengthen early detection, habitat management, and biodiversity protection, thereby offering essential baseline data for future conservation and management of local fish diversity.

## Simple Summary

Environmental DNA (eDNA) is a sensitive, non-invasive tool for detecting aquatic species, especially rare or invasive ones. In this first study of its kind in the Cuijiang River, we used eDNA metabarcoding—analysing DNA from water samples—to survey fish diversity. We detected 36 species, with Carassius being the most abundant, but also found invasive species like *Acipenser* and *Oncorhynchus* at notable levels, which could harm native fish. Fish diversity varied across sites, likely due to human activities and river connectivity. Our findings show that eDNA metabarcoding effectively complements conventional survey methods, particularly for early detection of elusive and invasive fish.

## 1. Introduction

As apex predators in aquatic food webs, fish communities exert profound influences on the structure and functioning of aquatic ecosystems [1,2]. Fish diversity is widely regarded as a critical indicator of ecosystem health, effectively reflecting the stability and overall condition of these systems [3,4,5]. Conventional approaches to assessing fish diversity—including fishery catch surveys, autonomous sampling, and underwater acoustic detection, among others—rely on direct observation and physical capture [6,7,8,9]. Nevertheless, these methods are often labor- and time-intensive, exhibit low detection sensitivity, suffer from substantial sampling biases, and may cause considerable disturbance to fish populations and their habitats [10,11,12]. In recent years, the advent of environmental DNA (eDNA) metabarcoding has introduced a novel and efficient paradigm for fish diversity survey and monitoring [13,14,15]. This technique involves extracting DNA from environmental samples, amplifying target genomic fragments via PCR, and subjecting the products to high-throughput sequencing, thereby enabling comprehensive community characterization [16,17,18]. Relative to traditional methodologies, eDNA metabarcoding not only enhances the speed and accuracy of species identification but also significantly reduces labor and material costs [19,20]. In aquatic environments, it has been successfully applied to evaluate fish diversity, providing a high-efficiency, non-invasive alternative to conventional capture-based approaches [21,22,23].

The efficacy of eDNA metabarcoding has been extensively validated across diverse aquatic systems. For biodiversity assessment, this approach has repeatedly demonstrated superior species detection capacity compared with conventional methods. For instance, studies employing universal primers recovered multiple species-specific sequences from water samples through multiple PCR replicates, detecting more fish species than nine concurrent conventional approaches [24]. Primers targeting the mitochondrial 12S rRNA gene successfully identified 168 of 180 marine fish species (93.3%) in an aquarium setting and detected 93 species in natural coral reef seawater, confirming the technique’s applicability in complex environments [25]. In Maizuru Bay, Japan, water samples collected within just 6 h yielded 128 fish species; of these, 40 species (62.5% of the 65 species recorded by visual census that had reference barcodes) had also been detected by a 14-year underwater visual census, while the eDNA method further revealed at least 23 additional species not observed by visual surveys, highlighting its unique capacity to uncover cryptic components of local fish assemblages [26]. eDNA technology has also demonstrated high sensitivity and early warning capability for invasive species monitoring. Since Ficetola et al. first applied eDNA to investigate the distribution of the American bullfrog (*Rana catesbeiana*), an invasive freshwater amphibian [27], the technique has been rapidly adopted for tracking invasive species dispersal. eDNA monitoring consistently outperforms conventional survey methods in detecting invasive aquatic species and delineating their distributions, as exemplified by the bullfrog (Lithobates catesbeianus) survey in France’s Périgord-Limousin Nature Reserve, where eDNA detected the species at 38 of 49 ponds (77.5%) versus only 7 (14.3%) by traditional auditory/visual encounters [28], with corroborating evidence from the Great Lakes for silver and bighead carps [29,30] and from Japan for bluegill sunfish [31].

The Xiangjiang River is a major tributary within the middle reaches of the Yangtze River system. Its first-order tributary, the Leishui River, flows northward through eastern Hunan Province. The Cuijiang River, located in the middle reach of the Leishui, serves as a key ecological corridor linking the upstream Dongjiang Lake with the mainstream of the Xiangjiang River. Hence, it is critically important to monitor and study fish diversity in the Cuijiang River. While extensive research has focused on fish diversity in the middle and lower Xiangjiang basin, and some protective measures have been adopted for the Cuijiang area in the upper reaches, a comprehensive and systematic assessment of its fish diversity is still lacking [32,33,34,35,36]. Conventional fishery surveys are inherently constrained by seasonal variations, limited site accessibility, and unavoidable disturbance to fish populations, which collectively render them unsuitable for high-frequency, spatially comprehensive, and sustained term monitoring [37,38,39]. Given the rapid pace of technological innovation, it is imperative to leverage novel methodologies for investigating fish diversity in the Cuijiang River. In this study, we employed environmental DNA (eDNA) metabarcoding to characterize fish diversity in the Cuijiang River. Our objectives were to understand the potential of eDNA-based approaches for fishery resource surveys in Cuijiang River and generate baseline data that will support future resource assessment and conservation efforts in this river system.

## 2. Materials and Methods

### 2.1. Sampling Site Location

**Figure 1:**
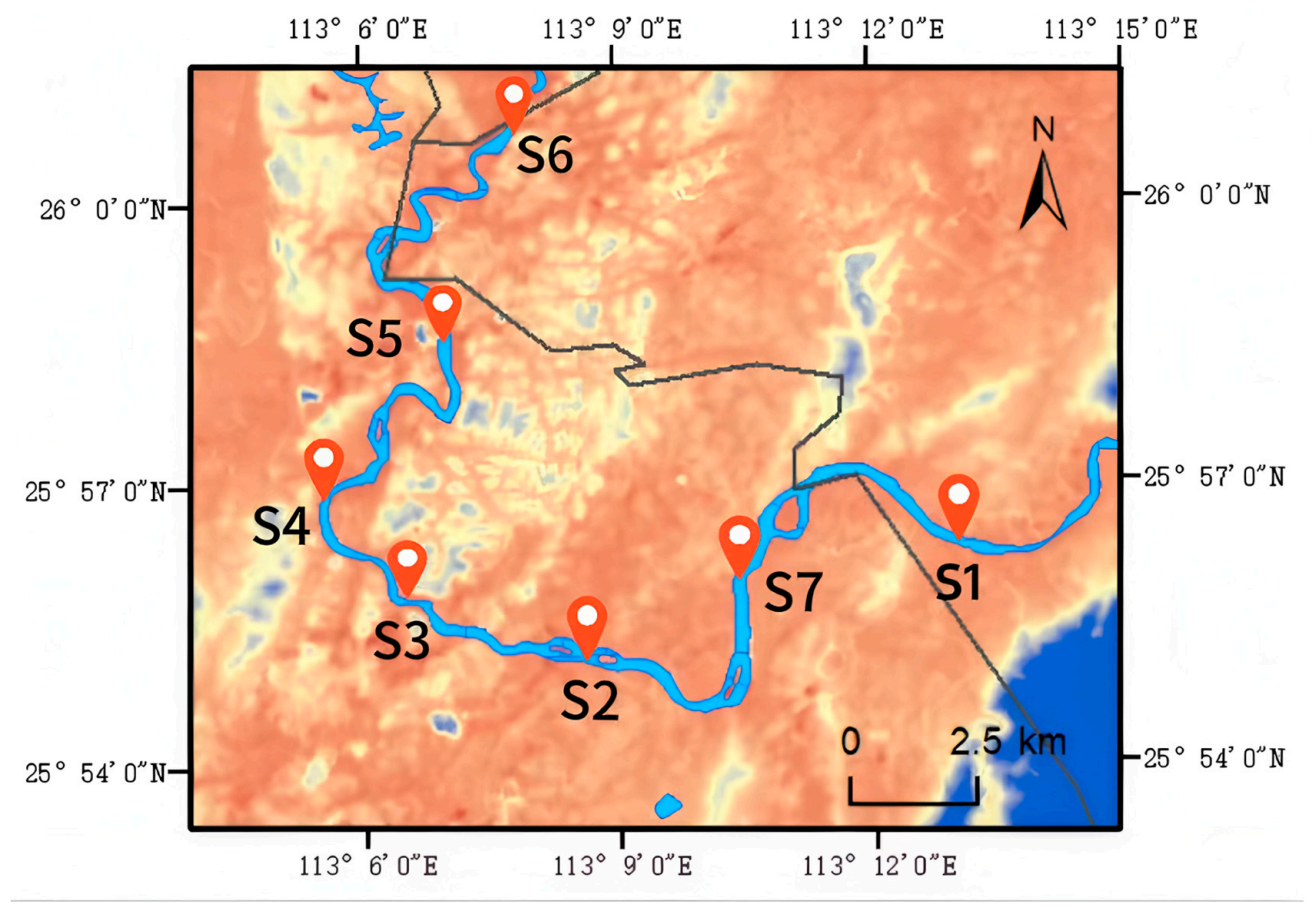
Layout of preset monitoring transects for water quality and aquatic ecology.

eDNA sampling for this study was carried out in October 2022. Along a roughly 31.114 km reach of the Cuijiang River mainstream—stretching from Chenzhou Dadao Bridge to Bianjiang Jinli Bridge—seven monitoring transects were delimited, taking into account variations in river length, topography, and the intensity of human activities. In alignment with these transects, seven representative sampling sites were established and coded as S1 through S7. Their spatial arrangements are illustrated in Figure 1.

### 2.2. Water Sample Collection and Processing

A total of 21 eDNA water samples were collected from seven sites. Sampling equipment comprised sterile 2 L bottles, a water sampler, 15 mL centrifuge tubes, and gloves. All collections were performed with gloved hands using a sterilized 2 L water sampler. For each sample, a composite water column from 0–5 m depth was obtained by lowering the sampler to 5 m and retrieving it slowly, thereby integrating water from different depths, including surface water below 30 cm, and then transferring the mixture into pre-treated bottles. At each site, triplicate samples were taken from two nearshore points and one mid-channel point. Following collection, all samples were immediately shielded from light and refrigerated.

### 2.3. Filtration of eDNA Water Samples and DNA Extraction

Prior to filtration, the vacuum filtration apparatus was thoroughly rinsed with ultrapure water, with particular attention paid to the membrane support area to prevent clogging. A procedural blank was prepared by filtering 2 L of ultrapure water under the same conditions. Water samples were then filtered through 0.45 μm mixed cellulose ester (MCE) membranes using a vacuum pump, and all filtration operations were carried out in a clean, draft-free environment to minimize airborne contamination. After each 2 L sample had been filtered, the apparatus was disassembled and rinsed again with ultrapure water. Using sterile forceps and wearing gloves, each membrane was carefully folded with the sample-bearing side inward, placed into an individual sterile 15 mL centrifuge tube, and stored at –20 °C. Following the completion of all filtrations, total DNA was extracted from the membranes using the QIAGEN DNeasy PowerWater Kit (Qiagen, Hilden, Germany), strictly following the manufacturer’s protocol.

### 2.4. PCR Amplification and Product Purification

In this study, the fish-specific primer pair AcMDB07 (forward: 5′-GCCTATATACCGCCGTCG-3′; reverse: 5′-GTACACTTACCATGTTACGACTT-3′), originally designed by Bylemans et al. to target the mitochondrial 12S rRNA gene, was used to amplify the extracted eDNA [40]. The expected amplicon length was 321 bp. PCR was conducted with 35 cycles of denaturation, annealing, and extension; the detailed reaction system and thermal cycling conditions are provided in Table 1a and Table 1b, respectively. Amplicons were stored at 4 °C after amplification. A negative control (ddH_2_O) was included, and three replicate PCRs were performed for each sample. The three replicate products were pooled and subjected to agarose gel electrophoresis. Subsequently, PCR products were purified by gel extraction using the AxyPrep DNA Gel Extraction Kit (AXYGEN, Union City, CA, USA). After completion of PCR amplification, the products were purified using magnetic beads, and their concentration was determined with a Qubit reader (Invitrogen (Thermo Fisher Scientific) Waltham, MA, USA). The size of the PCR products was verified by 1.5% agarose gel electrophoresis to confirm alignment with expected results. When the target product bands exhibited correct sizes and concentrations ≥ 2 ng/μL, further experiments could proceed. The purified amplicons were then subjected to high-throughput sequencing on the Nova-Seq PE250 platform.

### 2.5. Data Processing

Raw data from HTS were preprocessed as follows: First, all sequences underwent quality control using QIIME (v.1.9.1) to remove low-quality sequences (quality score < Q20, length < 100 bp, and those with mismatches), yielding high-quality valid sequences. Then, paired reads were assembled into complete sequences based on their overlapping regions. High-quality sequences for each sample were obtained using barcode adapters and primer sequences, with simultaneous correction of sequence orientation for each sample according to the direction of barcode adapters and primer sequences. OTUs were clustered using QIIME (v.1.9.1) software with a sequence similarity threshold of ≥97%. After clustering, the most abundant sequence in each OTU was selected as the representative sequence. These representative sequences were then aligned and annotated against the fish DNA barcode reference database in the NCBI-nt and MitoFish databases, generating the OTU abundance table for this dataset. It should be noted that the OTU read proportion was used solely to describe the composition of sequencing data and should not be interpreted as quantitative inference. Sequence reads that failed to match any species in duplicate samples from each sampling site were excluded, and the remaining species-matched sequence counts from each site were processed as mean values. Subsequently, based on the OTU clustering results, species composition analysis and annotation were performed to refine fish taxonomic information. Finally, Alpha diversity analysis (Shannon index, Simpson index, Chao1 index, ACE index, and Coverage) was conducted using the vegan package (v2.6-4) in R (v4.2.3).

## 3. Results

### 3.1. Fish Composition

Based on OTU assignments from eDNA metabarcoding, at least 36 fish species were detected in this reach, belonging to 6 orders, 13 families, and 30 genera. Among these, 22 species were reliably identified to the species level, and 35 fish taxa were resolved to the genus level. In addition, at least five non-native invasive species were recorded, including *Tinca tinca*, *Coptodon zillii*, and *Gambusia affinis*.

In the fish community, the order Cypriniformes was numerically dominant, comprising 3 families, 21 genera, and at least 25 species, accounting for 69.4% of the total taxa. Within this order, the family Cyprinidae contributed 18 genera and at least 21 species, Cobitidae contributed 2 genera and at least 4 species, and Balitoridae contributed 1 genus and 1 species. The second most abundant order was Perciformes, with 5 families, 5 genera, and at least 6 species, representing 16.67% of the total taxa; these included one genus each from the families Channidae, Serranidae, Cichlidae, Gobiidae, and Micropercops. Siluriformes comprised 2 families and at least 2 species, making up 5.26% of the total species, including one species from Siluridae and at least one from Bagridae. The remaining orders—Cyprinodontiformes, Salmoniformes, and Acipenseriformes—were each represented by a single family (Table 2).

### 3.2. Fish Community Structure

**Figure 2:**
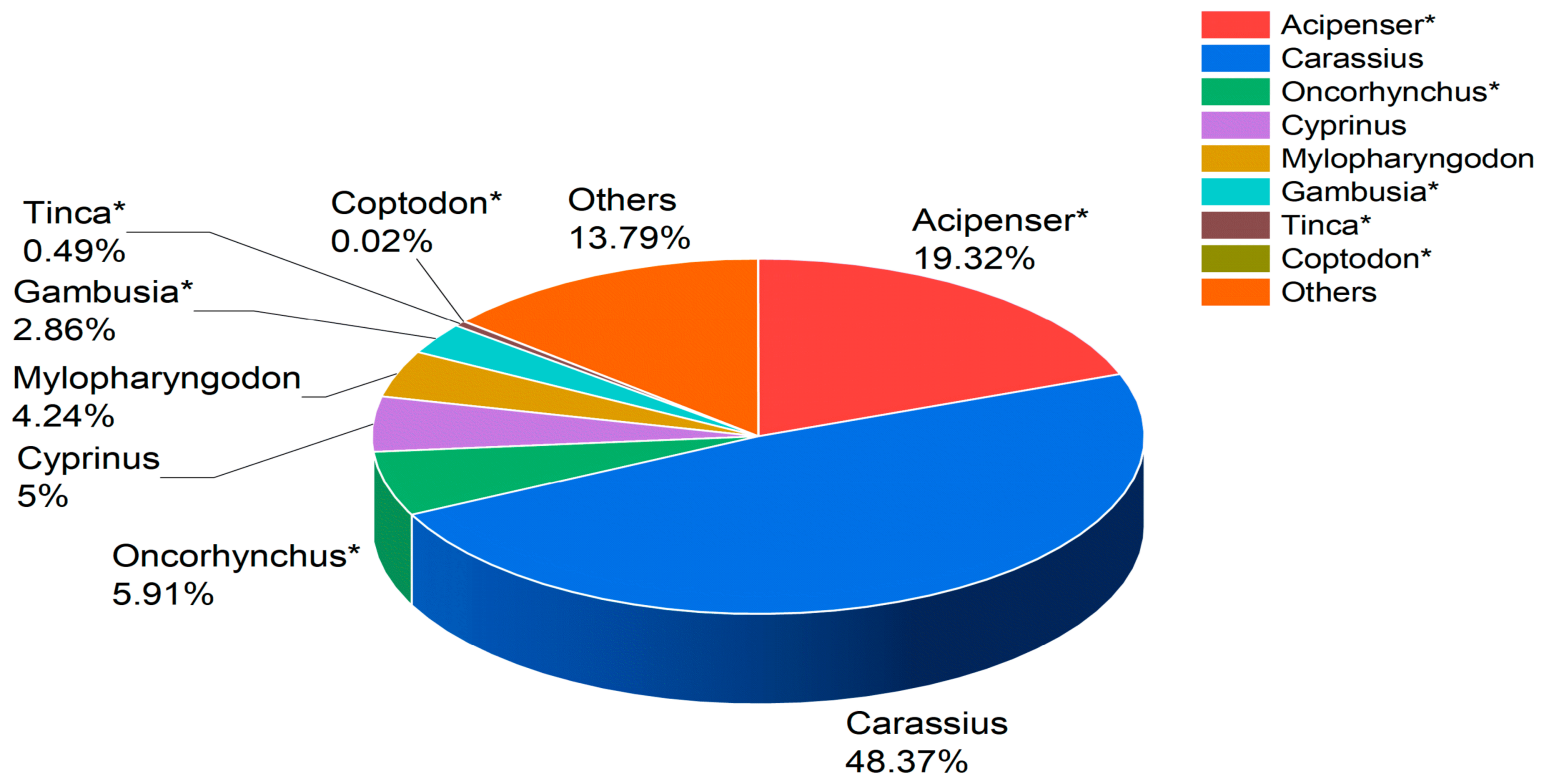
eDNA metabarcoding monitoring of OTU proportional composition in the target section of Cuijiang River (Note: * represents invasive alien species).

**Figure 3:**
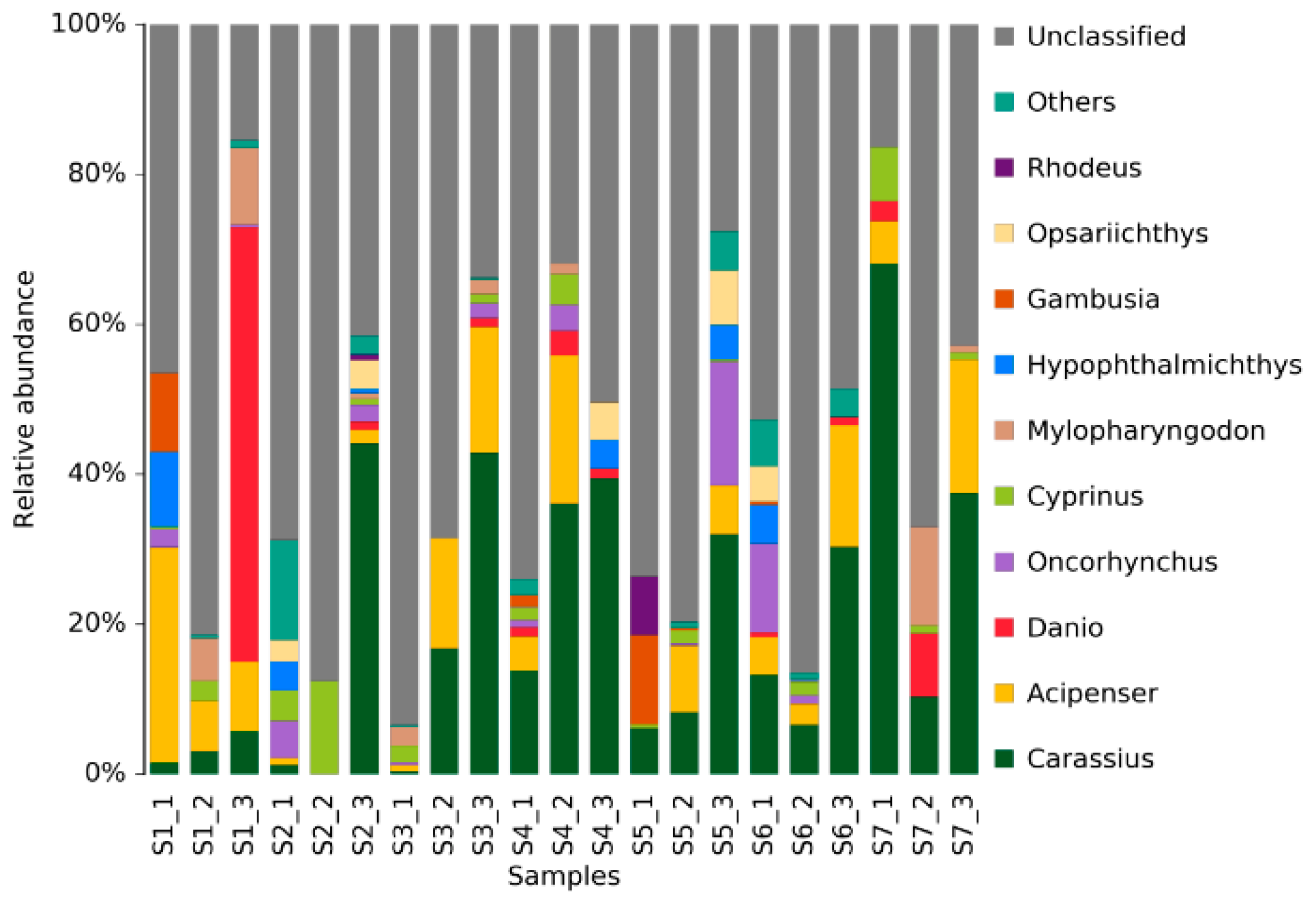
eDNA metabarcoding detection of genus composition at sampling sites in the target section of Cuijiang River.

Examination of the relative OTU abundances of the detected fish taxa showed that the five genera with the highest proportional representation were *Carassius*, *Acipenser*, *Oncorhynchus*, *Cyprinus*, and *Mylopharyngodon*, comprising 48.39%, 19.33%, 5.91%, 5.01%, and 4.24% of the total OTU abundance, respectively. *Carassius* ranked first in relative abundance among all genera. Furthermore, the aggregate relative abundance of OTUs attributable to invasive non-native species was markedly elevated, accounting for 28.61% of the total (Figure 2 and Figure 3).

Analysis of species detection across eDNA sampling sites revealed that the invasive genus *Acipenser* occurred in every environmental sample, corresponding to a detection frequency of 100%. Moreover, assessment of OTU sequence abundances across sites (Figure 3) indicated that non-native invasive species were widely distributed, being detected at multiple sampling points.

### 3.3. Fish Diversity

Estimation of four diversity metrics—Shannon, Simpson, Chao1, and ACE—demonstrated significant inter-site variation in the studied reach of the Cuijiang River basin. Site S3_3 showed the highest values for all indices, followed by S4_1 and S5_2, implying that the eDNA sampling effort was adequate to capture the local diversity. Additionally, coverage estimates ranged from 85.89% to 93.02% across sites, indicating that the sequencing depth was sufficient to recover the majority of OTUs and that the resultant data reliably represented the species composition of each sample (Table 3).

## 4. Discussion

### 4.1. Ecological Assessment of Fish Based on eDNA

Using eDNA metabarcoding, we surveyed fish diversity at seven sites along the studied reach of the Cuijiang River. A total of at least 36 fish species were detected, representing 6 orders, 13 families, and 30 genera (Table 2). The five genera with the highest proportional OTU abundances were *Carassius*, *Acipenser*, *Oncorhynchus*, *Cyprinus*, and *Mylopharyngodon*. The order Cypriniformes was the most diverse, comprising three families and at least 25 species, and was broadly distributed across sampling sites (Figure 3), underscoring its ecological significance in these freshwater systems. Many cypriniform species are omnivorous or herbivorous, thereby contributing to nutrient cycling, periphyton regulation, and energy flow across trophic levels [41,42]. Within the family Cyprinidae, species exhibit marked differences in their tolerance to environmental disturbance. As defined by the Fish Index of Biological Integrity (F-IBI) framework [5], cyprinids are positioned along a continuum from highly sensitive to highly tolerant, with *Carassius auratus* and *Cyprinus carpio* explicitly classified as tolerant species; elevated abundances of these taxa are positively associated with habitat degradation, organic pollution, and eutrophication [43,44]. In the Cuijiang River, *Carassius* dominated the fish community, and the invasive *Acipenser* also displayed a relatively high proportional abundance. Taken together, these patterns suggest that the river may be experiencing moderate disturbance or eutrophic conditions.

### 4.2. The Situation of Invasive Fish

Biological invasions are a major concern in conservation and management, given their roles in driving species loss, ecological degradation, and economic damage. The consistent detection of invasive fish across all environmental samples further corroborates the efficacy of our eDNA metabarcoding approach (Figure 3), which—consistent with previous studies on invasive species surveillance [25,26,27,28,29,30,31]—demonstrates strong capacity for alien fish detection. To explore the ubiquity of invasive fish at every sampling site, we posit that this pattern is primarily attributable to suboptimal biosecurity protocols at aquaculture operations along the river basin [45,46], in addition to unregulated anthropogenic stock enhancement practices [47,48]; these factors collectively promote the accidental introduction of exotic species into the river system. Among the detected invasive taxa, Oncorhynchus exhibited the highest proportional OTU abundance. Owing to its carnivorous feeding habits and wide ecological tolerance [49,50], Oncorhynchus has considerable potential to invade and occupy niches typically exploited by native species [51,52], thereby disrupting the established ecological equilibrium [53] and inducing population reductions in indigenous fishes [54]. Consequently, it is essential to implement targeted management actions to counteract the negative impacts of invasive fish on native biodiversity [55,56]. We recommend the following measures: (i) installation of physical containment and escape-prevention structures at aquaculture facilities around the Cuijiang River to curb the release of cultured exotics; (ii) establishment of routine invasive species monitoring programs to track their spatiotemporal trends and assess their ecological effects, thus enabling early warning and adaptive responses; and (iii) formulation of regulatory policies and public awareness campaigns to discourage unpermitted or indiscriminate fish releases into the river.

### 4.3. Spatial Variations in Fish Communities

Significant spatial variation was detected in the Shannon, Simpson, Chao1, and ACE indices across sampling sites in the target reach of the Cuijiang River basin (Table 3). Site S3_3 (the Chenjiang River estuary) recorded the highest values for all diversity metrics, followed by S4_1 (the downstream section of the Shimiantan Dam) and S5_2 (Dongjiang Bridge on Ziwu Road). These observed disparities in fish diversity among sites likely reflect differences in human activity intensity and land-use types [57,58,59], with diminished anthropogenic interference potentially favoring the maintenance and increase in fish diversity. Furthermore, the elevated diversity at S3_3 may be explained by its position at the confluence of the Chenjiang and Cuijiang rivers. Enhanced hydrological connectivity in such estuarine zones promotes fish movement and the widespread dispersal of eggs and larvae, which in turn influences species distributions and supports higher diversity levels [60,61,62,63].

### 4.4. Applicability and Limitations of eDNA Metabarcoding in Fish Species Detection

In this study, eDNA metabarcoding was applied to assess fish diversity within the target reach of the Cuijiang River. A total of at least 36 fish species were recorded in the basin, including 5 non-native invasive species and 31 native taxa. Notably, several small-bodied and infrequently recorded species (e.g., *Rhodeus ocellatus*, *Danio rerio*, and *Pseudorasbora parva*) were successfully identified (Table 2), confirming the elevated sensitivity of eDNA metabarcoding for small-sized and alien invasive taxa [64,65,66,67]. This heightened detectability is attributable to the method’s dependence on extracellular DNA released into the aquatic environment upon cell lysis, which enables the detection of species that are particularly challenging to capture via conventional approaches, especially those with small body sizes or sparse populations [68]. Nonetheless, eDNA technology has not yet rendered conventional field surveys or the expertise of professional taxonomists obsolete [37]. Despite its high sensitivity and low environmental impact, eDNA monitoring outcomes are subject to a variety of interfering variables. In the present study, a proportion of the detected taxa could be assigned only to the genus level. This limitation arises from a fundamental attribute of eDNA metabarcoding: its dependence on the amplification of short, predefined DNA fragments, which typically harbour limited genetic information [69,70,71]. In closely related species, sequence divergence across these short amplicons may be minimal or even absent, thereby reducing the power of species discrimination [72]. Moreover, even when the chosen barcode region is theoretically sufficient to distinguish two species, reliable identification can still be precluded by the lack of corresponding reference sequences in public databases such as GenBank [73,74,75]. In addition, the production and persistence of eDNA are modulated by multiple abiotic factors, including hydrological conditions, ultraviolet radiation, temperature, pH, and sediment properties [15,76]. Consequently, eDNA-based surveys may yield results that deviate to some extent from the true species composition and detection efficacy in situ.

Currently, eDNA metabarcoding is regarded as a semi-quantitative tool, offering distinct advantages and limitations in comparison with traditional monitoring methods [77,78]. Thus, depending on the specific research aims, the integration of both approaches not only permits the rapid characterization of fish diversity in the Cuijiang River basin but also provides opportunities for collecting samples to support complementary research endeavors.

## 5. Conclusions

Environmental DNA (eDNA) has established itself as a sensitive, non-invasive, and efficient alternative to traditional survey techniques, being particularly valuable for the early detection of rare and invasive taxa. The present study constitutes the first documented use of eDNA metabarcoding for fish diversity surveys in the Cuijiang River. In total, 36 fish species were detected, encompassing 6 orders, 13 families, and 30 genera. Among all sampling sites, Carassius showed the highest relative abundance, followed by the exotic invasive genera *Acipenser* and *Oncorhynchus*, which raises concern over their potential detrimental effects on native fish assemblages. Analyses of alpha diversity indicated inter-site heterogeneity in species richness, likely associated with anthropogenic disturbance and hydrological connectivity. Given that this is the first application of eDNA metabarcoding to fish community monitoring in the Cuijiang River, our results demonstrate the considerable potential of this molecular approach for freshwater ecosystem surveillance. Accordingly, we advocate its incorporation into current and future aquatic monitoring and conservation frameworks to strengthen early warning systems, habitat stewardship, and biodiversity safeguarding. However, the extensive adoption of eDNA technology is still hindered by several obstacles, notably the lack of comprehensive reference sequence libraries, vulnerability to contamination, detection efficacy, and the inherent difficulty of deriving reliable abundance estimates. Overcoming these hurdles will demand standardized protocols and the parallel deployment of conventional survey methods for cross-validation.

## Figures and Tables

**Table 1:** (**a**) Reaction system of the PCR amplification; (**b**) PCR amplification procedure.

**(a) Reaction system of the PCR amplification**	
**Reagents**	**Volume**
2 × Reaction Buffer for Taq DNA polymerase	15 μL
dNTPs (2.5 mmol/L)	1.5 μL
AcMDB07-F	1 μL
AcMDB07-R	1 μL
Taq DNA polymerase	1 μL
ddH_2_O	7.5 μL
Template DNA	3 μL
**(b) PCR amplification procedure**	
**Process**	**Time**
95 °C Initial denaturation	5 min
95 °C Denaturation	30 s
55 °C Annealing	30 s
72 °C Extension	45 s
72 °C Final extension	10 min

**Table 2:** Fish species composition detected in the target reach of Cuijiang River by eDNA metabarcoding.

Order	Family	Genus	Species
Cypriniformes	Cyprinidae	*Opsariichthys*	*O. bidens*
		*Mylopharyngodon*	*M. piceus*
		*Rhodeus*	*R. ocellatus*
		*Rhodeus*	Not identified
		*Acheilognathus*	Not identified
		*Acheilognathus*	Not identified
		*Acrossocheilus*	Not identified
		*Culter*	Not identified
		*Carassius*	*C. auratus*
		*Coreius*	*C. heterodon*
		*Cyprinus*	*C. carpio*
		*Hypophthalmichthys*	*H. molitrix*
		*Aristichthys*	*A. nobilis*
		*Mylopharyngodon*	*M. piceus*
		*Phoxinus*	Not identified
		*Xenocypris*	Not identified
		*Pseudorasbora*	*P. parva*
		*Danio*	*D. rerio*
		*Tinca*	* *T. tinca*
		*Saurogobio*	Not identified
	Cobitidae	*Misgurnus*	*M. bipartitus*
		*Misgurnus*	*M. mizolepis*
		*Misgurnus*	Not identified
		*Paramisgurnus*	*P. dabryanus*
	Balitoridae	*Schistura*	*S. fasciolata*
Perciformes	Channidae	*Channa*	Not identified
	Serranidae	*Siniperca*	Not identified
	Cichlidae	*Coptodon*	* *C. zillii*
	Gobiidae	*Rhinogobiu*	*R. giurinus*
		*Rhinogobiu*	*R. duospilus*
	Micropercops	*Micropercops*	*M. swinhonis*
Siluriformes	Bagridae	*Not identified*	Not identified
	Siluridae	*Silurus*	*S. meridionalis*
Cyprinodontiformes	Poeciliidae	*Gambusia*	* *G. affinis*
Salmoniformes	Salmonidae	*Oncorhynchus*	* Not identified
Acipenseriformes	Acipenseridae	*Acipenser*	* Not identified

Note: * represents invasive alien species.

**Table 3:** Alpha diversity indices of each sampling site.

Sample ID	Chao1	Shannon	Simpson	ACE
S1_1	45	2.9034	0.7986	45
S1_2	34	2.5611	0.6863	34
S1_3	47	2.5802	0.6681	47
S2_1	56	3.8706	0.852	56
S2_2	23	1.3972	0.4912	23
S2_3	53	2.7305	0.7122	53
S3_1	34	1.39	0.3754	34
S3_2	37	3.1711	0.8476	37
S3_3	90	3.2183	0.7772	90
S4_1	67	2.4752	0.5854	67
S4_2	45	2.9883	0.7848	45
S4_3	51	3.1289	0.7979	51
S5_1	59	2.0117	0.5335	59
S5_2	67	2.4599	0.5686	67
S5_3	53	3.5512	0.868	53
S6_1	51	3.9316	0.8887	51
S6_2	57	1.8905	0.4128	57
S6_3	36	2.3969	0.7212	37
S7_1	26	2.0543	0.5658	26
S7_2	30	2.2578	0.6865	30
S7_3	29	2.9645	0.7926	29

## Data Availability

The original contributions presented in this study are included in the article. Further inquiries can be directed to the corresponding author (zyshen@hunnu.edu.cn).
